# Monthly Summary

**Published:** 1870-12

**Authors:** 


### MONTHLY SUMMARY.

---

*Hygienic Information.*—As samples of the sort of trash which a confiding public is made to swallow under the name of hygienic information, one popular journal of health contained some time ago the startling announcement that to build houses with cellars underneath is a grave mistake, on account of the damp air which cellars contain (which would, of course, be entirely obviated by building directly on the ground); and in a later issue warns its readers against cutting their corns, on the ground that fatal and uncontrollable hæmorrhage is a frequent result of that operation. In another "health journal" a "Dr." Dio Lewis, of Boston, asserts that tomatoes cause a scorbutic condition of the gums, "a diseased action of the mucous lining of the alimentary canal," piles, and loosening of the teeth, so that they can be easily pulled out by the fingers; the latter inference being based on a case where a young woman, whose health had been failing for a year, was sent by her physicians advice into the country, where she "learned to eat tomatoes," and "almost immediately" her teeth became so loose that she was enabled to indulge in the amusement of removing about twenty of them. Of the periodicals dealing in sanitary information for popular reading, the only one that we have seen having the slightest pretensions to scientific accuracy is "*good health,*" and even this would be improved if some of its contributors would refrain from displaying too deep erudition in dogmatic statements concerning subjects which are still *subjudice.—Medical Gazette.*

---

*Strychnine as an Antidote for Chloral Poisoning.*—At a meeting of the Berlin Medical Society, in November, 1860, Dr. Liebriech communicated the result of his experiments in search of an antidote for chloral poisoning.

His investigations have not been directed to the discovery of a neutralizing agent for chloral while it remains in the alimentary canal, but after it had entered the circulation. The primary action of chloral being upon the brain and spinal cord, and its secondary upon the heart, he had sought for a remedy which had its immediate effect upon the latter organ.

It occurred to him that strychnine might meet this indication, which increased the force of the systole and lessened that of the diastole, the reverse effect of chloral. Experiments upon animals had confirmed its antidotal properties; its efficacy, however, being restricted to the profounder states of chloral asphyxia.

If merely hypnotic doses of chloral be given to an animal, the administration of strychnine will induce the usual tetanic phenomena. This does not occur where the dose of chloral has been sufficient to affect the function of the spinal cord.

The fact was shown by the following experiments: A fatal dose of chloral was given to a rabbit, and death shortly ensued. The same quantity was given to a second, but as the respiration began to flag, a fatal dose of strychnine was injected (hvpodermically). It soon roused from stupor, and perfectly recovered.

Two days afterward the same quantity of strychnine was again given, and death occurred in fifteen minutes. He therefore advises its use where the life of the individual is threatened from over-doses of chloral, at the same time employing the ordinary irritants and excitants.

During the discussion which followed the reading of the report, Dr. Von Langenbeck suggested that it would be difficult to determine the quantity of strychnine to be used subcutaneously in asphyxiated states from either chloroform or chloral.

Dr. L., from his experiments upon animals, estimates the dose at about 10 milligr. for adult individuals.—*Memorabile Heilbronn. Chicago Medical Examiner.*

---

*Death from Chloroform.*—Dr. George Johnson, in a letter to the Editor of the London *Medical Times and Gazette,* writes as follows:—

"Dr. Richardson, in his lecture on death from chloroform, referring to the *post-mortem* appearances in cases of what he designates epileptiform syncope, states that the lungs are blanched and bloodless, the left side of the heart and the systemic arteries empty; while the right side of the heart and the whole systemic venous system are distended. Dr. Richardson explains the appearances thus:—'All through the body there is evidence on the arterial side of the circulation of intense arterial contraction. * * * * the arteries have poured their contents into the veins, and contracting firmly in their minute ramifications have shut up the blood in the veins, and produced complete arrest of motion throughout the circulation.'

"I find a difficulty in accepting this explanation of the facts. It must be borne in mind that the contraction of the minute muscular arteries has a stop-cock, and not a force-pump action, an obstructive and not a propulsive influence upon the circulation. Contraction of the minute ramifications of the systemic arteries would retain the blood in the arterial trunks, and not drive it on into the veins; and, again, driving the blood into the veins would not arrest the circulation if the blood were free to move through the lungs. The condition of the heart, lungs, and bloodvessels, which occurs in animals rapidly killed by chloroform, and which is also found in cases of acute apnœa, and of cholera collapse, appears to me to admit of only one explanation. Contraction of the minute pulmonary arteries arrests the blood before it reaches the capillaries. The closure of the arterial stop-cocks in the lungs tends to empty the vessels in front, and to distend those which are behind the seat of obstruction; hence, anæmia of the lungs, and emptiness of the left side of the heart, and the systemic arteries, while the trunks of the pulmonary artery, the right cavities of the heart, and the systemic veins, are distended."

---

*Removal of the Articular Extremity of the Lower Jaw with Restoration of Motion.*—Mr. Christopher Heath, University College Hospital, London, and Dr. J. E. Garretson, of this city, both record cases of the removal of the articular extremity of the lower jaw with complete restoration of motion after a few months. In Dr. Heath's case the condyle was removed on the 16th of June, 1869, and in December the movements are reported to be as perfect as though no disease existed.

In Dr. Garretson's case the full left half of the bone was removed in January, 1866. The case was lost sight of about one month after the operation, and seen two years later, when motion was found perfect. Both operations were for necrosis.—*Med. and Surg. Reporter.*

---

*Loss of Speech After Chloroform*—The *London Lancet* quotes from a German periodical the statement that a servant girl inhaled chloroform for the purpose of having a tooth extracted. On awaking she had lost the power of speech, could not utter any sound whatever, and remained in that state for five weeks, in spite of various remedies, especially electricity. After this time she began to speak in a low tone, and was put under appropriate treatment. It is supposed that she suffered during anæsthesia from rupture of some cerebral vessel. She had never been hysterical.

---

*Iodide of Potassium in Periostitis.*—Mr. Paget, in a clinical lecture at St. Bartholomew's, advises, in treating periostitis, repeated blisters. Iodine of potassium is useful, 1, in syphilitic periostitis; 2. less so in gouty periostitis; 3, less in scrofulous; 4, it has no power in chronic rheumatic arthritis.—*Med. Record.*

*Neuralgia Pill.*—In the January number of the New Orleans *Journal of Medicine*, Dr. Osborn, of Greensboro, Ala., publishes the following formula, which, in his practice, he has found very effectual in the relief of neuralgic affections:

| | | |
|---|---|---|
| ℞ | Zinci cyanidi | gr. vj. |
| | Quiniæ sulphatis | gr. ix. |
| | Morphiæ sulphatis | gr. iss. |
| | Extracti belladonnæ | gr. iij. |

M. et ft. mas. div. in pil No. vj. Sig. One pill every 6 honrs till pain is relieved.

---

*Vehicel of Exhibiting Chloral Hydrate.*—Dr. Napheys, in his "Modern Therapeutics" gives this formula:

| | | |
|---|---|---|
| ℞ | Chloral hydratis, | ℥ ss. |
| | Syrup Tolutani, | |
| | Aquæ, | aa. f. ℥iii. M. |

Sig.—One to four tablespoonful for dose with water.

---

*Carbolic Acid Soap.*—A pharmaceutist sends the following formula to the *Drug. Circ. and Chem. Gazette* for preparing the *Sapo Desinficiens phenylatus Hageri:* Take freshly prepared cocoa-nut oil soap, 150 parts, and fuse; then add a solution of—

Alcohol, 10 parts; carbolic acid, 6 parts; caustic potassa, 2 parts; oil of lemon, 1 part, and mix with stirring. To be poured into moulds.

---

*Poisoning by Worm Lozenges.*—Dr. B. D. Gifford reports to the Boston *Med. and Surg. Journal* a case of poisoning of a child three years old, who had eaten seven "worm lozenges." The symptoms were those of strychnia, and indications of the presence of strychnia were found in lozenges from the same parcel. The vermicidal agent was supposed to be santonin. In the *U. S. Dispensatory* a similar case is related which occurred in France nine years ago. The patient was poisoned by what was considered an overdose of santonin, but strychnia was afterwards detected. How came the strychnia in the two parcels of santonin? Or was there some other toxic element developed in the manufacture of the santonin resembling strychnia in its physiological and chemical relations?—*Am. Eclectic Med. Review.*

